# Research on pharyngeal bacterial flora in transoral atlantoaxial operation and the postoperative follow-up study

**DOI:** 10.1186/s12891-022-05851-3

**Published:** 2022-10-19

**Authors:** Yu Zhang, Suo-Chao Fu, You Wu, Chu-Song Zhou, Xiang-Yang Ma

**Affiliations:** 1 Department of Orthopaedics, General Hospital of Southern Theatre Command of PLA, Guangzhou, 510010 Guangdong People’s Republic of China; 2 Department of Orthopaedics, Zhu-Jiang Hospital of Southern Medical University (First Military Medical University), Guangzhou, 510282 Guangdong People’s Republic of China

**Keywords:** Craniovertebral junction, Transoral approach, Infection prevention, Spinal lesion, Prospective study

## Abstract

**Objectives:**

To study the changes of bacterial flora after a series of preoperative oral disinfection and the postoperative recovery of nerve function of patients with craniovertebral junction disorders who were treated with transoral approach operations.

**Methods:**

This research analyzed 20 cases collected from October 2009 to May 2010. All these patients were with CVJ disorders, including 8 males and 12 females, aged 2 to 66 (38.1 on average), and they were all treated with transoral approach operations. The mucosa samples of the posterior pharyngeal wall were sent for bacteria culture. These samples were collected by sterile cotton swabs at four crucial points, including 3 days before operation/before gargling, 3 days after continuous gargling/after anesthesia intubation on the day of operation, after intraoperative cleaning and washing of the mouth, and after intraoperative iodophor immersion. The microflora was stained by means of smear and further counted after an investigation by microscope. The neural function of patients was evaluated by the ASIA classification and the JOA scores. All patients but two with posterior stabilization performed respectively underwent transoral atlantoaxial reduction plate (TARP) fixation consecutively in the same sitting. A regular reexamination of cervical vertebra with lateral and open mouth X-ray, CT and MRI was conducted after operation to evaluate the reduction of atlantoaxial dislocation, internal fixation position, bone graft fusion, inflammatory lesions and tumor recurrence.

**Results:**

This bacteriological research showed that the mucosa of the posterior pharyngeal wall of all the patients was in a sterile state after a series of oral preoperative preparations and intraoperative iodophor disinfection, which was considered as type I incision. The bacterial culture results of the mucosa samples of the posterior pharyngeal wall collected at different time points showed significant differences (χ^2^ = 42.762, *P* = 0.000). All the patients had improvement in ASIA, and their neural functions were improved to different levels after operation. There was a significant difference in JOA scores before and after operation (*t* = 8.677, *P* = 0.000). Postoperative imaging examination showed that the atlantoaxial screw position was good and firm, and the CVJ disorders were treated appropriately.

**Conclusion:**

It is safe and effective to cut the posterior pharyngeal muscle layer and implant internal fixation by means of transoral approach.

## Introduction

The craniovertebral junction (CVJ)—defined as the occiput, atlas, and axis—is a complex area that houses vital neural and vascular structures. It represents the ultimate link between the head and spine with its absolute need for structural support as well as mobility. The majority of the spinal rotation, flexion, and extension occur between the occiput, the atlas, and axis. Because it is an area with dense, overlapping and complex anatomical structures, surgical intervention for trauma, deformity, tumor or inflammation in this area is extremely challenging. Due to these factors, many spinal surgeons have paid great attention to the basic surgical and clinical research on this area [1–4].

In the past 30 years, many scholars have conducted extensive and in-depth research on its anatomy, biomechanics, injury and developmental deformity pathology. And they tried to find out safe, simple and practical surgical techniques to achieve decompression, stability and functional reconstruction. However, anatomic abnormalities caused by old injuries and severe complex deformities in this area, especially atlantoaxial dislocation, fracture deformity of occipital or atlantoaxial, inflammatory or tumorous erosion, have brought great difficulties and risks to surgical treatment [5, 6].

The transoral approach is the preferred midline approach for ventral CVJ extradural lesions, because it is performed in a midline plane that is relatively avascular and parallels the course of the cranial nerves at the CVJ. Furthermore, it provides the most direct route to osseous and soft-tissue abnormalities that are ventral to the brainstem and therefore it provides the shortest working distance for lesion treatment. The transoral approach can be used for the surgical treatment of various diseases, including congenital or secondary malformation (basilar invagination [7–9], atlas assimilation [10, 11], os odontoideum [12], atlantoaxial fixed dislocation [13]), trauma [14], tumor [15], spondylotic [16], autoimmune (most frequently rheumatoid arthritis [17, 18]), chronic inflammatory diseases [19], and osteomyelitis [20].

However, some scholars query the infection rate of transoral approach operations. They hold the opinion that factors such as the weak alkaline saliva and food residue in the oral cavity, and the connection to nasal cavity and pharynx provide an appropriate condition for a large number of bacteria to multiply [21, 22].

Considering the simultaneous implantation of internal fixations, they consider that the incidence of the incision infection after transoral approach operations may be much higher than that of posterior operations. However, that’s not the truth. Since 1984, the author’s department of Orthopaedics has treated more than 1000 patients with CVJ disorders by means of transoral approach (over 700 operations performed in the author’s hospital and over 300 consultation operations performed in other hospitals). During this long-term process, the hospital has continuously explored and improved relevant operative techniques, and summarized a set of perioperative treatment plans, which laid a solid foundation for the successful implementation of the operations [7, 9, 14]. This article aims to study the oral bacterial flora situation of patients with CVJ disorders before their operations, during the preparation process and after disinfection. By adopting the experimental methods of microbiology, this study provides the experimental basis for the research on the prevention of postoperative infection and perioperative safety in terms of transoral approach operations.

## Materials and methods

### Patient clinical data

All patients with anterior lesions compressing the cervicomedullary tract who underwent transoral surgery from October 2009 to May 2010 were included in this study. Radiographic imaging, previous and postoperative evaluations as well as outpatient archives were checked. Thirteen patients presented CVJ malformation (odontoid basilar invagination, atlas assimilation, os odontoideum, atlantoaxial dislocation), 4 were trauma victims, 1 presented rheumatoid arthritis, 1 lesions were tumoral and 1 pathologies were eosinophilic granuloma. None of the above cases had a history of diabetes. The mean age at the time of treatment was 38.1 years (range 2 ~ 66 years); 12 females and 8 males were included in this study. Selection criteria [8]: Candidates must have no local infection in their oral cavity before operation, no dental caries, no upper respiratory infection, or no history of antibiotic use in the past 3 months. Moreover, it’s also compulsory for them to be suitable for transoral approach operation. Local pain referred as cervical and/or cranial pain was present in 20 patients. Manifest quadriparesis of different grade was detected in 18 cases (motor/sensory). Cerebellovestibular signs were detected in nine patients. The lower cranial nerves were affected in 12 patients. Pathognomic physical marks such as short neck, low hairline and facial asymmetry were observed in 10 cases of CVJ malformation. Duration of symptoms at admission was 4 days to 23 years. According to the classification of American Spinal Injury Association (ASIA)，there were 4 cases of Grade B, 8 of Grade C, 6 of Grade D, and the other 2 of Grade E. The Japanese Orthopedic Association (JOA) scores ranged from 6 to 16, with an average of 10.30 ± 2.96. All these evaluations were carried out preoperatively. Atlantoaxial mobility was checked by means of dynamic X-ray (atlantodental interval, clivodental interval, spinal canal diameter) in cases of CVJ malformation [8, 9]. MRI and 3D-CT scan were used to detect the site of compression, the extension of the lesion (in case of tumor or rheumatoid pannus, gadolinium contrast was used), to determine the position of the vertebral artery (in order to prepare the posterior stabilization). The indications for surgery were: tumor, compressing pannus, irreducible compression (fracture, malformation), tip of the odontoid process at least 2.5 mm above the Chamberlain’s line. Standard transoral approach was used in 20 patients, odontoidectomy was required in 1 patient (platybasia,odontoid tip more than 20 mm above Chamberlain’s line) and one case required palate split. All patients but two with posterior stabilization performed respectively underwent transoral atlantoaxial reduction plate (TARP) fixation consecutively in the same sitting.

### Methods

#### Volunteer recruitment and sampling

Bacteria associated with the mucosal surfaces of the pharyngeal wall were collected from twenty volunteers above (age range, 2–66 years; gender, 8 M, 12 F). The sampling procedure was as follows [23, 24]. Sterile cotton swabs (Technical Services Consultant Ltd), pre-moistened in PBS (pH 7.4) were inserted into the oral cavity until reaching the centre of the posterior pharyngeal wall [23]. Representative samples were derived through gentle axial rotation of the swab (10s). Tongue compressors were used to prevent contamination of the sample by the tongue microbiota. Three swabs were collected from each site at four crucial points, including 3 days before operation/before gargling, 3 days after continuous gargling/after anesthesia intubation on the day of operation, after intraoperative cleaning and washing of the mouth, and after intraoperative iodophor immersion respectively. The specimens of the posterior pharyngeal wall were sent by specially-assigned staff for examination immediately after their collection.

#### Bacterial culture of the samples

Considering the common pathogenic bacteria in the upper respiratory tract (nasal cavity, nasopharynx and throat) including Streptococcus and Neisseria [23]. Experimenters inoculated the samples on the blood agar plate, chocolate plate and McConkey agar plate. These samples were then cultured in a 35 °C incubator with 5% CO_2_ for 24 hours. The microflora was stained by means of smear and further counted for drug sensitivity tests after an investigation by microscope [24].

Oropharyngeal counts were recorded as colony forming units (c.f.u. mm^− 2^). Briefly, the sample area of swabs was determined as follows. Tips of swabs were immersed in crystal violet (1%, v/v) and impressions made on paper. Once dried, the surface areas of the imprints were recorded and a mean total sampling area calculated. A standardized procedure for all throat sampling was used to minimize variation [24]. The collection of oropharyngeal swabs from volunteers was approved by the Hospital ethics Committee on the Ethics of Research on Human Beings (ref TPCS/ethics/201917).

### Evaluation index

The nerve function of patients before and after operation was evaluated by the American Spinal Injury Association (ASIA) classification and the Japanese Orthopedic Association (JOA) scores. A regular reexamination of cervical vertebra with lateral and open mouth X-ray, CT and MRI was conducted after operation to evaluate the reduction of atlantoaxial dislocation, internal fixation position, bone graft fusion, inflammatory lesion and tumor resection in the craniovertebral junction.

### Statistical analyses

The quantitative data are presented as mean and standard deviation. The statistical significance of the differences among the experimental groups was evaluated. By means of SPSS25.0, Chi-Square (χ^2^) test was conducted to examine the bacterial producibility of each sample, and Paired-Samples T Test was performed according to the before and after operation JOA scores. Value was *P ≤* 0.05 was considered significant.

## Results

### Bacterial culture results of samples

Table 1 illustrates the cultured species present on the oropharyngeal wall mucosa of twenty individuals with transoral operation at different time points. The complex communities developed in vitro were compared to direct oropharyngeal swabs through the principal components analysis of the binary band matching profiles generated from stable microcosms. It can be seen that significant differences exist among the four time points (χ^2^ = 42.762, *P* = 0.000). Stable microcosms were clustered within different groups, exhibiting significant homology to previous reports [23]. Sequence analysis of major resolved bands suggested posterior pharyngeal wall microcosms to be colonized by *Streptococcus mitis* and Neisseria spp. These two bacterias were also found to be dramatically decreased (10^7^ to 10^5^ or 10^5^ to 10^3^) after 3 days of gargle and intubation. In addition，several bacterias were reported，including 3 cases of *klebsiella pneumoniae* (10^5^ ~ 10^7^)，1 case of streptococcus constellatus subspecies (10^7^), and 1 case of sarcomella mucilage (10^5^). This may result from bacterial contamination of nasal cavity or endotracheal tube which is caused by anesthesia intubation. However, after intraoperative cleaning and gargling，the types and quantity of bacteria cultured in the samples which were collected in the oral cavity continued to decrease to 10^3^ or a few bacteria-free ones compared with those obtained after 3 days of anesthesia intubation. Besides, no bacterial growth was found in all the 20 samples taken from the posterior pharyngeal wall after iodov immersion disinfection, indicating that the mucosa at the incision of the posterior pharyngeal wall was in a sterile state.

**Table 1:** Bacterial culture of posterior pharyngeal wall microbiotas at different time points(*n* = 3)

Dwelling time point	Oropharyngeal microbiotas					
	*Streptococcus mitis* and Neisseria spp.	*Streptococcus mitis* or Neisseria spp.	*Klebsiella pneumoniae*	Subspecies of *Streptococcus constellatus*	*Serratia marcescens*	Abacterial growth
T1	10^5^ ~ 10^7^ (9 cases)	10^5^ ~ 10^7^ (4 cases)	0	0	0	0 (7 cases)
T2	10^3^ ~ 10^5^ (9 cases)	10^5^ (6 cases)	10^5^ ~ 10^7^ (3 cases)	10^7^ (1 case)	10^5^ (1 case)	0
T3	10^3^ (1 case)	10^3^ (10 cases)	0	10^3^ (1 case)	< 10^5^ (1 case)	0 (7 cases)
T4	0	0	0	0	0	0 (20 cases)

Three days before operation/before gargling (T1); Three days after continuous gargling/after anesthesia intubation on the day of operation (T2); after intraoperative cleaning and washing of the mouth (T3); after intraoperative iodophor immersion (T4)

### Surgical and radiographic analysis

**Fig. 1:**
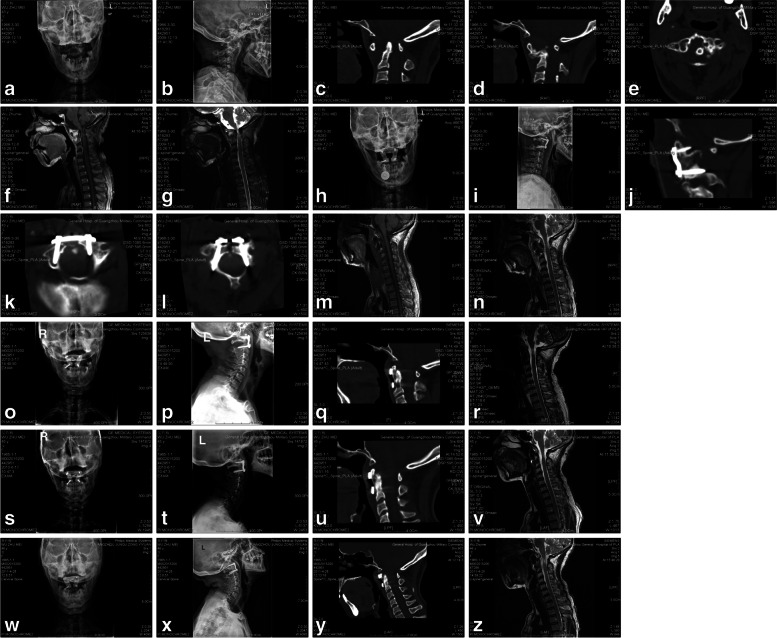
Shows the examination results of a 43-year-old female with basilar invagination, atlantoaxial dislocation and neurofibromatosis. She was treated with transoral atlantoaxial reduction, antogenous iliac bone-grafting and internal fixation with the help of transoral atlantoaxial reduction plate. As shown in **a** and **b**, the X-rays reflect obvious anterior atlantoaxial dislocation. The sagittal CT (see **c**) further shows that odontoid tip is 2.5 mm higher than the Chamberlain line, which can be considered as basilar invagination. According to **d** and **e**, the sagittal and axial CTs (near the left lateral mass) show callus hyperplasia in the atlanto-dental interval (ADI) space, leading to the irreducible atlantoaxial dislocation. MRI results (see **f** and **g**) show significant compression of cervical medulla at atlantoaxial level and partial degeneration of spinal cord. The postoperative X-rays (see **h** and **i**) immediately show that the atlantoaxial vertebra has been completely reducible, and the internal fixation is in good position. According to **j**, **k** and **l**, the postoperative CTs immediately show good position of the atlantoaxial screw path, no invasion of vertebral artery and spinal canal, and the bony callus in the ADI space is removed. The postoperative MRI results (see **m** and **n**) demonstrate that there is no significant compression of cervical spinal cord at atlantoaxial level, and no significant changes in partial degeneration of spinal cord. X-rays (see **o** and **p**) conducted 3 months after the operation show that the internal screw fixed in the atlantoaxial segment is in place without obvious loosening or displacement. The CT (see **q**) shows callus formation in the ADI, and initial osseous fusion of the atlantoaxial segment. The MRI (see **r**) shows that there is no significant compression of cervical spinal cord and no significant change in partial degeneration of spinal cord. X-rays which were conducted 6 months (see **s** and **t**) and 17 months (see **w** and **x**) after the operation demonstrate that the atlantoaxial internal fixation is firm without any obvious dislocation. In addition, the physiological curve of cervical spine is good, and there is no instability of adjacent segments. The CTs conducted 6 months (see **u**) and 17 months after operation (see **y**) show further callus formation in the ADI space, and firm osseous fusion. The MRIs conducted 6 months (see **v**) and 17 months (see **z**) after operation show no significant compression of cervical spinal cord. In addition, partial degeneration of spinal cord shows some improvement

**Fig. 2:**
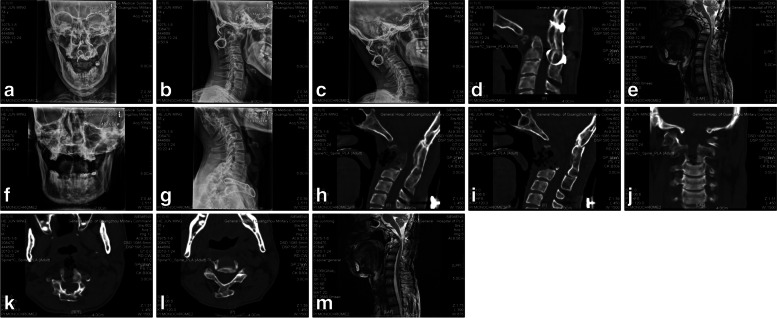
Shows the examination results of a 35-year-old male with the malunion after the thrice operations of atlantoaxial dislocation. He was treated with posterior occipitoatlantoaxial cable removal followed by second-stage transoral C2 corpectomy. X-rays (see **a**, **b** and **c**) show space occupying of steer wire internal fixation and good posterior plate fusion in occipital and atlantoaxial segments, without any instability or displacement. The CT results (see **d**) reveal firm posterior plate fusion in occipital and atlantoaxial segments, with Brooks steer wires fixed inside. Moreover, the sagittal diameter of the spinal canal is markedly narrow. MRI results (see **e**) show significant compression and partial degeneration of spinal cord. As shown in **f** and **g**, the inside steer wires of occipital and atlantoaxial segments have been removed completely. Besides, the posterior bone plate is of structural integrity without obvious fracture or dislocation. The CT results (see **h**, **i**, **j**, **k** and **l**) show the odontoid process of C2 vertebra has been removed, with only a small amount of bone residue. MRI results (see **m**) show that the compression of spinal cord at atlantoaxial level is significantly less than that before operation. The sagittal diameter of spinal canal is effectively restored. Moreover, the flow of cerebrospinal fluid in the posterior dural sac is obviously unobstructed, and there is no significant change in the partial degeneration of the spinal cord

**Fig. 3:**
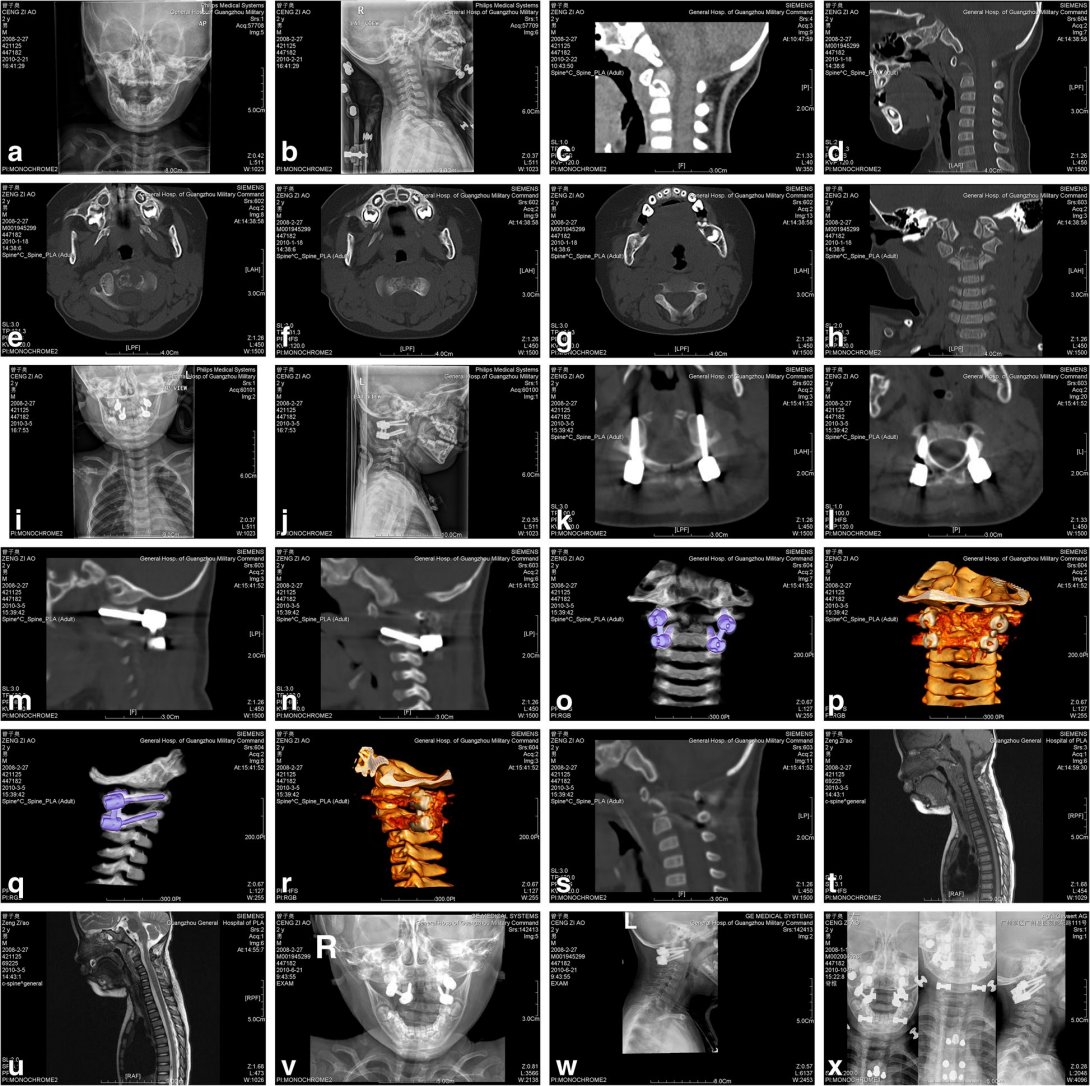
Represents the examination results of a 2-year-old male with eosinophilic granuloma of odontoid process accompanied by atlantoaxial dislocation. He was treated with poterior C1/2 pedicle screw internal fixation followed by transoral lesion resection of odontoid process. The X-rays (see **a** and **b**) show anterior atlantoaxial dislocation. As shown in **c** and **d**, the CT further reveals atlantoaxial dislocation, bone destruction in odontoid process of C2, and space occupying lesions. According to **e**, **f**, **g** and **h**, CTs show the lesions are mainly distributed in the odontoid process and lumbar region of C2, and no obvious lesions are found in the basal region. The postoperative X-rays (see **i** and **j**) immediately show that the atlantoaxial segment has been completely reducible, and the internal fixation is in good position. As shown in **k**, **l**, **m**, **n**, **o**, **p**, **q**, **r** and **s**, the atlantoaxial segment has been repositioned, and all the screw paths are in good position, without any invasion of vertebral artery holes and spinal canals. The postoperative MRI results (see **t** and **u**) demonstrate the odontoid lesion at atlantoaxial level has been removed. According to **v** and **w**, X-rays conducted 3 months after operation show that the internal screw fixed in atlantoaxial segment is in place without obvious loosening or displacement. X-rays (see **x**) conducted 6 months after operation demonstrate that the atlantoaxial internal fixation is firm without any obvious dislocation. In addition, the physiological curve of cervical spine is good, and there is no instability of adjacent segments

**Fig. 4:**
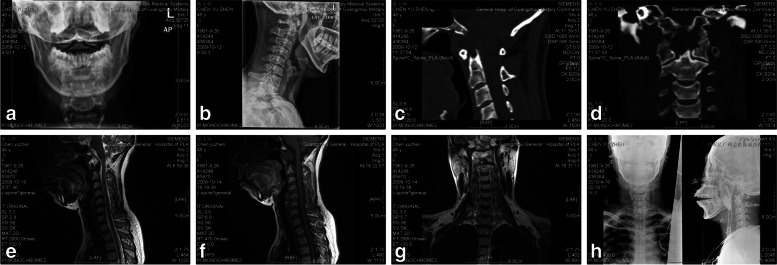
Illustrates the inspection results of a 49-year-old female with atlantoaxial chordoma. She was treated with transoral chordoma resection of C2. According to **a** and **b**, the X-rays have shown bone destruction in the odontoid process of axis, but without any obvious dislocation. Figure **c** and **d** represent the sagittal and coronal CT results. These figures further illustrate bone destruction in the odontoid process of C2 and space occupying lesions. MRI results (as shown in **e**, **f** and **g**) reflect the space occupying lesions in the odontoid process of axis. As shown in **h**, the postoperative X-rays immediately show that odontoid process lesions have been removed

After their successful operations, all the 20 patients underwent X-ray of lateral and open-mouth cervical spine, CT scan of atlantoaxial spine and MRI of cervical spine for reexamination immediately after operation. These twenty operations include 17 cases of transoral atlantoaxial reduction, antogenous iliac bone-grafting, and internal fixation with the help of third-generation transoral atlantoaxial reduction plate (TARP-III) (as shown in Fig. 1), 1 case of posterior occipitoatlantoaxial cable removal followed by second-stage transoral C2 corpectomy (as shown in Fig. 2), 1 case of poterior atlantoaxial pedicle screw internal fixation followed by transoral lesion resection of odontoid process (as shown in Fig. 3) and 1 case of transoral chordoma resection of C2 (as shown in Fig. 4). All these 20 operations last 132 ~ 445 min (258.55 ± 79.87 min), and the intraoperative blood loss for each operation amounts to 50 ~ 2000 ml (244.00 ± 431.38 ml). There is no intraperative injury in vertebral artery and spinal cold, no postoperative plate loosening, or no relevant complications such as incision infection and intracranial infection. There is only 1 case of giant polyp in the posterior pharyngeal wall (local polypectomy was performed after the E.N.T. consultation). Patients have their symptoms such as neck pain, limb numbness and weakness, etc., improved to different degrees after the operation. The patients were followed up for 2 to 23 months, with an average of 5.15 ± 5.26 months. Their neurological symptoms also showed significant improvement (showed in Table 2). Among the 4 cases which were classified into ASIA Grade B before operation, 3 cases were improved to Grade C, while the rest remained unchangeable. All the 8 cases with ASIA Grade C and 6 cases with Grade D were improved by grade 1 to 2. The average preoperative JOA scores were 10.30 ± 2.96 points, while the scores were improved to 13.35 ± 1.90 points 3 months after operation. There is a significant difference before and after operation (*t* = 8.677，*P* = 0.000).

**Table 2:** The Classification of ASIA of patients

No.	1	2	3	4	5	6	7	8	9	10	11	12	13	14	15	16	17	18	19	20
a	D	D	C	D	B	C	D	C	C	D	C	C	E	B	D	C	B	C	B	E
b	D	D	D	D	C	D	D	D	C	D	D	D	E	C	D	D	C	C	B	E

a: ASIA of before surgery

b: ASIA of after surgery

## Discussions

Currently, a number of 3A hospitals in our country have adopted transoral approach operations to treat craniovertebral junction disorders. However, the lack of familiarity or understanding of this approach limits the clinical thinking of clinicians. Many spine surgery professionals have been worried about the safety of transoral approach operations because of the typeIIincision and the infection of incision after transoral fixation implantation. Due to the high risk and high mortality [25, 26] of anatomical structure of craniovertebral junction, internal fixation in oral cavity is considered as a forbidden zone for doctors.

This is related to the long-term lack of systematic research and understanding of transoral bacteriology. To solve the above problems, especially to get more recognition and promotion of transoral approach from spine surgery professionals, we adopt a series of preoperative and intraoperative cleaning and disinfection measures [27, 28] and further transform type II incision with relative bacteria in the posterior pharyngeal wall into typeIsterile incision through strict bacteriological evaluation research so as to ensure the safe and effective implementation of transoral internal fixation. Although the number of 20 patients enrolled in this experiment is comparatively small which requires further comprehensive and systematic experimental design and more follow-ups, the successful experience of surgery also reflects that strict preoperative and intraoperative cleaning and disinfection process is effective and safe.

Over the past decade, thousands of transoral approach operations have been performed in our hospital, through which we have accumulated rich clinical experience in surgical treatment of craniovertebral junction disorders. Moreover, our hospital has successfully treated patients with difficult and complex diseases, such as adult/infant congenital basilar depression [7], refractory/irreversible atlantoaxial dislocation [9, 29] and atlantoaxial tumor [30] or inflammation occupying lesion [31], and further improved and summarized relevant key points of operations [32], such as the classification and surgical treatment of developmental canal stenosis at atlas level [33], axial reverse pedicle screw placement [34, 35] and atlantoaxial posterior combined screw placement [9, 31].

Our experience in oral cleaning preparation before transoral approach operations is as follows. (1) Patients would receive the consultation of department of stomatology after they are admitted to the hospital. If they are found to have oral diseases with symptoms, such as obvious dental caries, gingivitis, sinusitis, etc., they will not be allowed to have the operation under that condition. They can only have the operation after the successful oral treatment. If they do not have such diseases, their teeth will be cleaned immediately after admission. (2) Oral cavity preparation would start 3 days before the operation. Patients will be asked to rinse their mouths with chlorhexidine acetate (or chlorhexidine) four times a day. (3) Patients will not be allowed to have the operations if they have any colds or other upper respiratory tract infection symptoms. In addition to the above oral cavity preparation, the following preoperative preparations should be paid attention to for the smooth operations: ① It’s compulsory to check if the patient’s mouth opening is large enough. Generally, patients need to open their mouth as large as possible under the non-anesthetic state, and the at least 4 cm space between the incisor and lower central incisor is enough for operation. ② Nasogastric tube would be indwelled preoperatively for the postoperative nasal feeding. ③Patients are required to have preoperative imaging examinations, including open-mouth and lateral X-rays, CT or CTA, and MRI or MRA. ④ If 3D printing rapid prototyping is available to make the model, the atlantoaxial vertebra and craniovertebral junction can be printed 1:1 into the model (including the important anatomical structures such as vertebral artery walking). After high-temperature and high-pressure disinfection, it would be taken to the operating table to facilitate the intraoperative guidance of screw placement and other key operations [5, 29].

## Conclusions

In conclusion, according to the bacteriology research, it is found that typeII incision with relative bacteria in the posterior pharyngeal wall could been transformed into typeIsterile incision in the transoral approach operations after a series of preoperative preparation and intraoperative disinfection of oral cavity. It is completely safe and effective to cut the posterior pharyngeal muscular layer open and implant an internal fixator by means of transoral approach. Important operations like the decompression and reconstruction of stable structures can be achieved at the same time period. It is proved that transoral approach is an optional method to treat craniovertebral junction disorders. Moreover, the spinal surgeons’ clinical treatment thinking has been effectively expanded by undergoing TARP operations.

## Data Availability

The data used and analyzed during the current study are available in anonymized form from the corresponding author on reasonable request.

## Abbreviations

TARP: Transoral atlantoaxial reduction plate
ADI: Atlantodental interval
JOA: Japanese Orthopaedic Association
ASIA: American Spinal Injury Association
CT: Computed tomography
MRI: Magnetic resonance imaging
